# Nomogram Model for Predicting the Prognosis of High-Grade Glioma in Adults Receiving Standard Treatment: A Retrospective Cohort Study

**DOI:** 10.3390/jcm12010196

**Published:** 2022-12-27

**Authors:** Peng Du, Xionggang Yang, Li Shen, Jiawei Chen, Xiao Liu, Xuefan Wu, Aihong Cao, Daoying Geng

**Affiliations:** 1 Department of Radiology, Huashan Hospital, Fudan University, Shanghai 200040, China; 2 Department of Radiology, The Second Affiliated Hospital of Xuzhou Medical University, Xuzhou 221000, China; 3 Department of Orthopedic Surgery, Huashan Hospital, Fudan University, Shanghai 200040, China; 4 Department of Radiology, Jiahui International Hospital, Shanghai 200233, China; 5 Department of Neurosurgery, Huashan Hospital, Fudan University, Shanghai 200040, China; 6 School of Computer and Information Technology, Beijing Jiaotong University, Beijing 100044, China; 7 Department of Radiology, Shanghai Gamma Hospital, Shanghai 200040, China

**Keywords:** nomogram model, adult high-grade glioma, progression-free survival, overall survival

## Abstract

Objectives: To identify the critical factors associated with the progression-free survival (PFS) and overall survival (OS) of high-grade glioma (HGG) in adults who have received standard treatment and establish a novel graphical nomogram and an online dynamic nomogram. Patients and Methods: This is a retrospective study of adult HGG patients receiving standard treatment (surgery, postoperative radiotherapy, and temozolomide (TMZ) chemotherapy) at Huashan Hospital, Fudan University between January 2017 and December 2019. We used uni- and multi-variable COX models to identify the significant prognostic factors for PFS and OS. Based on the significant predictors, graphical and online nomograms were established. Results: A total of 246 patients were enrolled in the study based on the inclusion criteria. The average PFS and OS were 22.99 ± 11.43 and 30.51 ± 13.73 months, respectively. According to the multi-variable COX model, age, extent of resection (EOR), and IDH mutation were associated with PFS and OS, while edema index (EI) was relevant to PFS. In addition, patients with IDH and TERT promoter co-mutations had longer PFSs and OSs, and no apparent survival benefit was found in the long-cycle TMZ adjuvant chemotherapy compared with the standard Stupp protocol. Based on these critical factors, a graphical nomogram and online nomogram were developed for predicting PFS and OS, respectively. The calibration curve showed favorable consistency between the predicted and actual survival rates. C-index and time-dependent AUC showed good discrimination abilities. Conclusions: We identified the significant predictors for the PFS and OS of HGG adults receiving standard treatment and established user-friendly nomogram models to assist neurosurgeons in optimizing clinical management and treatment strategies.

## 1. Introduction

Glioma is the most common primary malignant tumor of the adult central nervous system (CNS). Due to its invasive growth, most patients will recur even after combined treatments such as surgery, radiotherapy, chemotherapy, targeted therapy, and immunotherapy [1]. The latest edition of the WHO classification of tumors of the CNS in 2021 divided gliomas into grades 1–4, from low to high grade [2]. Most adult gliomas are high-grade, fast-growing, and aggressive. The current standard treatment for adult HGG is surgery within the maximum safety range, followed by radiotherapy and concurrent temozolomide (TMZ) chemotherapy, and six cycles of TMZ adjuvant chemotherapy (Stupp protocol) [3]. Despite standardized treatment, the median survival of HGG patients was only 14.6 months [4].

Surgery is the basis of the standard treatment for glioma, which is closely related to the prognoses of patients [5,6]. Multiple retrospective studies and large-scale meta-analyses have shown that extended surgical resection can significantly prolong the progression-free survival (PFS) and overall survival (OS) of glioma patients compared with partial resection or biopsy [7,8,9,10,11]. In addition, radiotherapy plays an important role in the treatment of glioma and can kill or inhibit residual tumor cells and prolong the survival of patients [12,13,14]. In addition, TMZ is currently the first-line single-agent chemotherapy drug for glioma, with the advantages of low toxicity and strong anti-tumor activity [15,16]. However, there has been controversy over the optimal number of cycles of postoperative TMZ adjuvant chemotherapy.

IDH is an important indicator of glioma molecular classification, which plays a significant role in the diagnosis, individualized treatment, and prognosis of gliomas [17]. Studies have shown that IDH-wildtype gliomas are more prone to recur than IDH-mutated gliomas [18]. MGMT is a DNA repair enzyme and is mainly distributed in the cytoplasm, repairing DNA to maintain the stability of the genome in cells. If the MGMT promoter is methylated, it will cause the loss of MGMT expression, resulting in a decrease in DNA repair and making gliomas more sensitive to chemotherapy drugs such as TMZ [19,20]. It is generally believed that patients with methylation of the MGMT promoter respond better to TMZ treatment [21]. 

We included both the grade three and four gliomas in the analysis, as they are all managed with the same treatment in China (the Stupp protocol). In order to identify the critical factors associated with the prognosis of HGG patients who have received standard treatment, we retrospectively analyzed the relevant data of patients, including basic information, tumor resection extent, tumor grade and genotyping, the interval between surgery and radiotherapy, postoperative TMZ adjuvant chemotherapy cycles, and radiological data. Furthermore, a novel web-based individualized survival prediction calculator was also developed and validated for these patients.

## 2. Patients and Methods

### 2.1. Patients 

This retrospective study was approved by the institutional review board of Huashan Hospital, Fudan University. The requirement for written informed consent was waived. The data of patients with HGG who received standard treatment at Huashan Hospital, Fudan University between January 2017 and December 2019 were reviewed. Eligible criteria were as follows: (1) patients aged above 18 years; (2) preoperative KPS ≥ 60; (3) HGGs confirmed by histopathology (grades 3 and 4); (4) no previous treatment history of intracranial tumors; (5) patient received standard treatment (surgery, postoperative radiotherapy, and TMZ chemotherapy); (6) no other concurrent treatments; (7) no progression or death occurred during the standard treatment cycle and at the completion of treatment; (8) complete radiological data before and after standard treatment; and (9) complete clinical data and genetic testing results. 

### 2.2. Patients’ Information

The collected patient information included the following two aspects: (1) clinical variables: sex, age, KPS (preoperative), tumor resection extent, tumor grade, IDH mutation, MGMT promoter methylation, TERT promoter mutation, interval between surgery and radiotherapy, and postoperative TMZ adjuvant chemotherapy cycles; (2) radiological variables (preoperative): tumor volume, maximum diameter, edema index (EI), T1 weighted imaging (T1WI), T2 weighted imaging (T2WI), fluid-attenuated inversion recovery (FLAIR), diffusion-weighted imaging (DWI), and enhanced pattern. The follow-up deadline for this study was 1 September 2021.

### 2.3. Treatment 

#### 2.3.1. Surgery

All patients received tumor resection surgery and the criterion was the removal of the tumor as completely as possible while preserving organ function. The extent of the tumor resection was evaluated according to a pre- and postoperative brain MRI, which was divided into total resection (>95%), subtotal resection (80–95%), and partial resection (<80%).

#### 2.3.2. Radiotherapy

All patients received radiotherapy after surgery. With reference to the patient’s pre- and postoperative radiological data, the target volume was delineated on the CT images of the treatment planning system, including the gross tumor volume (GTV), clinical target volume (CTV), and at-risk organs (spinal cord, brain stem, eyeball, lens, optic nerve, optic chiasm, eyeball, etc.). The total dose of the target volume was 54–60 Gy, divided into 27–30 times, and each divided dose was 1.8–2.0 Gy, 5 times a week for 5–6 weeks. 

#### 2.3.3. Chemotherapy

All patients took TMZ orally (75 mg/m^2^·d) at the same time during radiotherapy, and TMZ adjuvant chemotherapy was given four weeks after radiotherapy, with a dose of (150–200 mg/m^2^·d) used every 28 days for 5 days (one cycle). The dose of the first cycle was 150 mg/m^2^·d, and the dose of the second cycle and later was 200 mg/m^2^·d. A portion of patients took 6 cycles of TMZ (Stupp protocol), and the other patients took more than 9 cycles of TMZ (long-cycle protocol). 

### 2.4. Follow-Up and Efficacy Evaluation

A brain MRI before surgery was used as a baseline for judging the efficacy of subsequent treatment or tumor progression. A brain MRI every 2 months after surgery was used for follow-up. All MRI examinations were acquired using a 1.5T MRI system (SIGNA Excite HD; GE Healthcare, Milwaukee, WI, USA). MRI sequences included T1WI, CE-T1WI, T2WI, FLAIR, and DWI, and the total acquisition time per patient was approximately 24 min. RANO criteria were used to evaluate the treatment response [22]. The progression-free survival (PFS) was defined as the time from the date of surgery to the first radiology-confirmation of intracranial disease progression or tumor recurrence. Overall survival (OS) was defined as the time interval from surgery to last follow-up or to death.

### 2.5. Statistical Analysis

The continuous data including tumor volume, maximum diameter, and EI were transformed into dichotomous variables, utilizing the optimal cut-off points based on running log-rank tests, and all variables were finally presented as numbers and percentages. 

All patients were randomly divided into the training sample and validation sample, with a ratio of 7:3. Then, a univariable COX analysis (based on the “survival” package) was conducted for all predictors to screen out the potentially significant prognostic factors (*p* < 0.15), which were included in the multivariable COX model (based on the “survival” package). Using the significant factors with the multivariable model, a novel graphical nomogram (based on the “rms” package) and a dynamic online nomogram (based on the “DynNom” package), were both generated. The calibration curve, time-dependent AUC (the area under the ROC curve) curve, and C-index were all plotted/calculated.

All statistical analyses were carried out using R version 4.1.3, and statistical significance was set at a probability value of 0.05.

## 3. Results 

### 3.1. Baseline Characteristics

A total of 246 patients were enrolled in this study based on the inclusion criteria, 172/74 of which were divided into the training and validation samples, respectively. There were 162 grade 4 gliomas and 84 grade 3 gliomas. Of these, 35 grade 3 gliomas presented with IDH1 mutations. The baseline characteristics are summarized in Table 1. The average PFS and OS were 22.99 ± 11.43 and 30.51 ± 13.73, respectively. The running Log-rank test of PFS indicated that the optimal cut-off points for EI, maximum diameter, and tumor volume were 3.09, 1.64 cm, and 1737.15 cm^3^, respectively (Supplementary Material Figure S1), and the running Log-rank test of OS indicated that the optimal cut-off points for EI, maximum diameter, and tumor volume were 3.09, 1.37 cm, and 1419.04 cm^3^, respectively (Supplementary Material Figure S2).

### 3.2. Univaraible and Multivariable COX Analyses

The results of the univariable analysis are shown in Table 1. 

The significant factors associated with PFS include the extent of resection (EOR) (HR = 1.373, 95%CI: 1.111–1.697, *p* = 0.003), IDH mutation (HR = 0.266, 95%CI: 0.177–0.402, *p* < 0.001), MGMT, promoter methylation (HR = 0.515, 95%CI: 0.345–0.769, *p* = 0.001), TERT promoter mutation (HR = 0.596; 95%CI: 0.408–0.870; *p* = 0.007), and EI (HR = 1.582, 95%CI: 1.005–2.490, *p* = 0.047).

The DWI sequence signal (*p* = 0.132) was found to be marginally associated with PFS.

**Figure 1:**
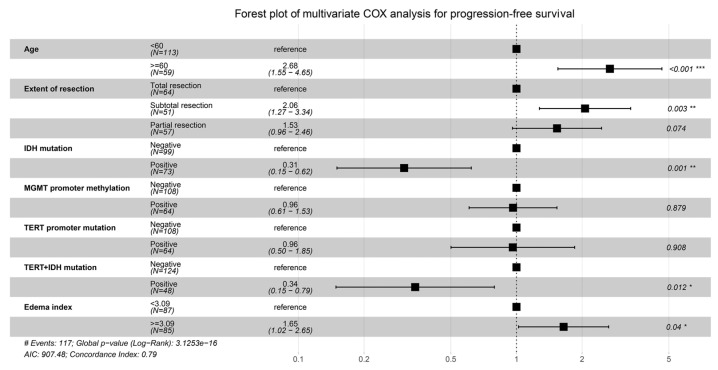
Forest plot for the multivariable COX analysis of PFS. Age, extent of resection, IDH mutation, IDH and TERT promoter co-mutation, and edema index were identified as the significant prognostic factors for PFS. * *p* < 0.05,** *p* < 0.01, *** *p* < 0.001.

**Figure 2:**
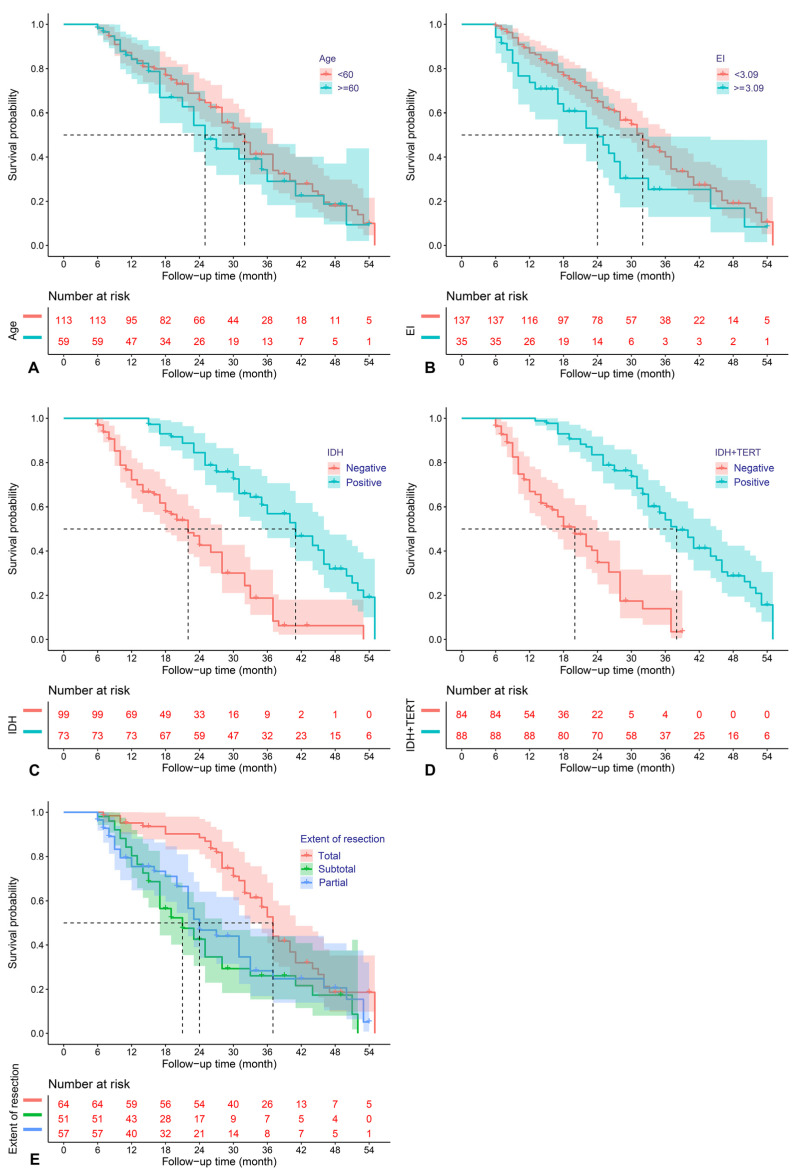
Kaplan–Meier survival curves for age (**A**), edema index (**B**), IDH mutation (**C**), IDH and TERT promoter co-mutation (**D**), and extent of resection (**E**), which were significantly associated with PFS in the multivariable analysis.

The above predictors were then analyzed utilizing a multivariable COX model, and the results are presented in a forest plot (Figure 1). Age (HR = 2.68; 95%CI: 1.55–4.65; *p* < 0.001), EOR (HR = 2.06; 95%CI: 1.27–3.34; *p* = 0.003), IDH mutation (HR = 0.31; 95%CI: 0.15–0.62; *p* = 0.001), IDH and TERT promoter co-mutation (HR = 0.34; 95%CI: 0.15–0.79; *p* = 0.012), and EI (HR = 1.65; 95%CI: 1.02–2.65; *p* = 0.04) were finally identified as the independent prognostic factors for PFS. The Kaplan–Meier survival curves for the five significant factors are plotted in Figure 2.

The significant factors relevant to the OS include EOR (HR = 1.305, 95%CI: 1.036–1.643, *p* = 0.024), IDH mutation (HR = 0.339, 95%CI: 0.218–0.525, *p* < 0.001), MGMT promoter methylation (HR = 0.609, 95%CI: 0.400–0.928, *p* = 0.021), and TERT promoter mutation (HR = 0.650; 95%CI: 0.434–0.974; *p* = 0.037). The Flair sequence signal (*p* = 0.126) was found to be marginally relevant to OS.

**Figure 3:**
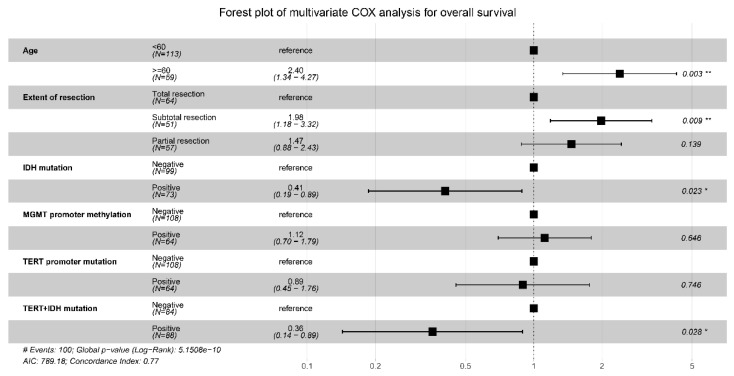
Forest plot for the multivariable COX analysis of OS. Age, extent of resection, IDH mutation, and IDH and TERT promoter co-mutation were identified as the significant prognostic factors for OS. * *p* < 0.05,** *p* < 0.01.

**Figure 4:**
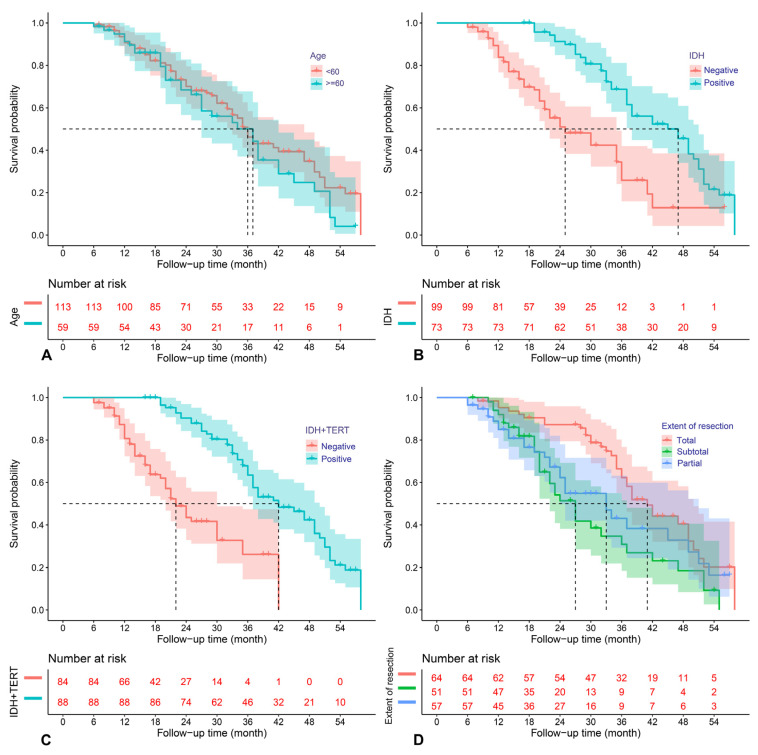
Kaplan–Meier survival curves for age (**A**), IDH mutation (**B**), IDH and TERT promoter co-mutation (**C**), and extent of resection (**D**), which were significantly associated with OS in the multivariable analysis.

The above predictors were then analyzed using the multivariable COX model, and the results are presented in a forest plot (Figure 3). Age (HR = 2.40; 95%CI: 1.34–4.27; *p* = 0.014), EOR (subtotal vs. total resection: HR = 1.98; 95%CI: 1.18–3.32; *p* =0.009), IDH mutation (HR = 0.41; 95%CI: 0.19–0.89; *p* = 0.023), and IDH and TERT promoter co-mutation (HR = 0.36; 95%CI: 0.14–0.89; *p* = 0.028) were finally identified as the independent prognostic factors for OS. The Kaplan–Meier survival curves for the four significant factors are plotted in Figure 4. 

### 3.3. Establishment and Validation of the Nomogram Model

**Figure 5:**
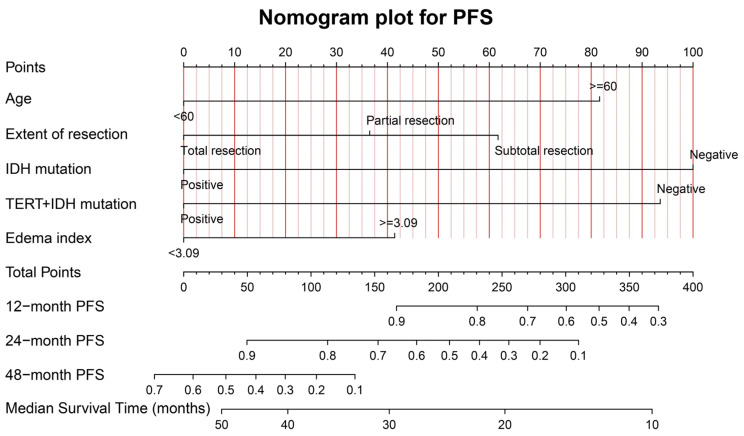
The graphical nomogram model for PFS based on the five significant predictors. The model could provide the predicted PFS at 12, 24, and 48 months, and the median survival time.

Figure 5 shows the graphical nomogram model including the five independent prognostic indicators for PFS selected using the multivariable COX model, which could predict the patients’ 12-, 24-, and 48-month survival and median survival time.

The calibration curves for PFS at 12, 24, and 48 months for the training (A–C) and validation (D–F) samples, are shown in Supplementary Material Figure S3. The favorable consistency between the predicted and actual survival rates are presented. The C-indexes are 0.79 (95%CI: 0.72–0.86) and 0.68 (95%CI: 0.49–0.87) for the training and validation samples, respectively. The time-dependent AUC curves for the training and validation samples are presented in Supplementary Material Figure S4. The moderate discrimination ability of the novel model is presented according to the AUC curve. 

**Figure 6:**
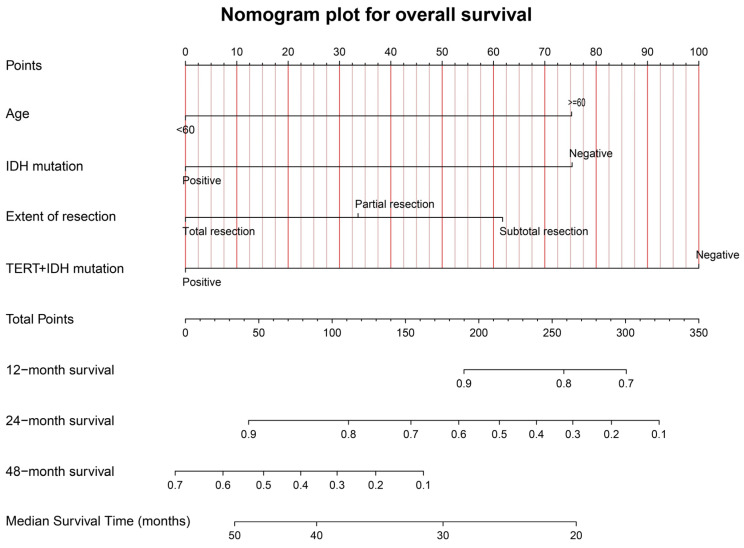
The graphical nomogram model for OS based on the four significant predictors. The model could provide the predicted survival rates at 12, 24, and 48 months, and the median survival time.

Figure 6 shows the graphical nomogram model including the four independent prognostic indicators for OS selected utilizing the multivariable COX model, which could predict the patients’ 12-, 24-, and 48-month survival and median survival time.

The calibration curves for OS at 12, 24, and 48 months, for the training (A–C) and validation (D–F) samples, are shown in Supplementary Material Figure S5. The favorable consistency between the predicted and actual survival rates are presented. The C-indexes are 0.77 (95%CI: 0.69–0.85) and 0.71 (95%CI: 0.55–0.87) for the training and validation samples, respectively. The time-dependent AUC curves for the training and validation samples are presented in Supplementary Material Figure S6. The moderate discrimination ability of the novel model is presented according to the AUC curve. 

### 3.4. Online Dynamic Nomogram Model Establishment

**Figure 7:**
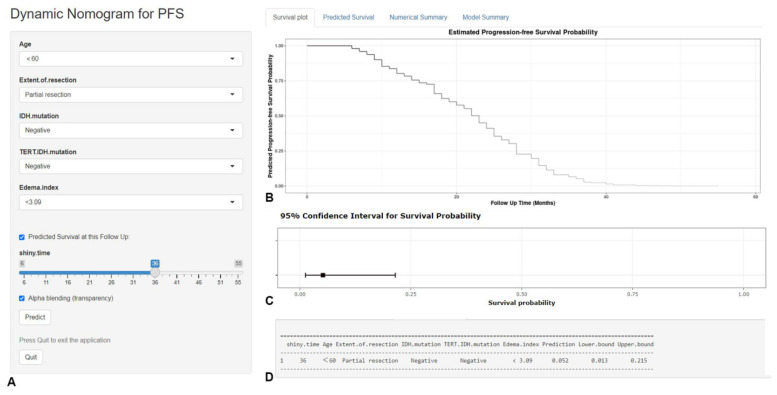
The screenshot of the online dynamic nomogram for PFS. After designating the items (**A**), neurosurgeons could easily plot the survival curve for an individual patient (**B**), predict the survival rate at each time point (**C**), and view the model summary (**D**).

**Figure 8:**
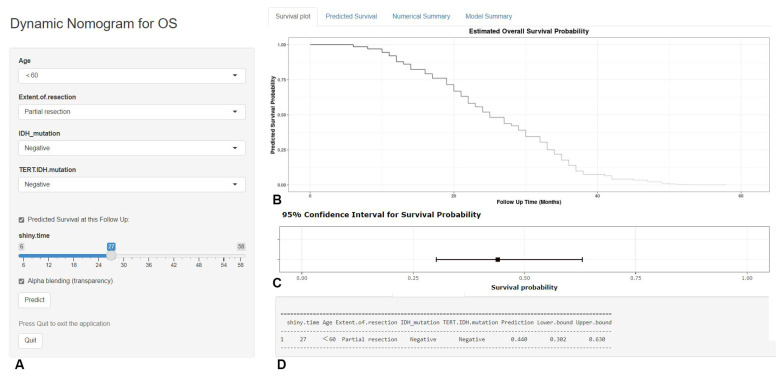
The screenshot of the online dynamic nomogram for OS. After designating the items (**A**), neurosurgeons could easily plot the survival curve for an individual patient (**B**), predict the survival rate at each time point (**C**), and view the model summary (**D**).

Based on the predictors screened by the multivariable COX model and the graphical nomogram model, the online dynamic nomogram models for PFS (https://glioma.shinyapps.io/survival_prediction_tool/, access data: 10 November 2022) and OS (https://glioma.shinyapps.io/survival_prediction_tool_os/, access data: 10 November 2022) were then established. The neurosurgeons could easily plot the survival curve for each patient and predict the survival rate at each time point. The screenshot of the online prediction tool is available in Figure 7 and Figure 8, respectively.

## 4. Discussion

Glioma is the most common primary CNS tumor originating from glial cells, accounting for 80% of primary intracranial tumors [23]. The clinical characteristics of HGG are highly malignant and prone to recurrence, which makes the treatment very tough. In recent years, molecular detection of glioma has achieved a certain degree of development, including MGMT promoter methylation, co-deletion of 1p/19q, IDH mutation, TERT promoter mutation, EGFR amplification, etc. This molecular information plays an important role in the prognosis and treatment of glioma [24,25,26]. In the WHO classification of tumors of the CNS in 2021, the integration of histopathology and molecular classification makes the diagnoses more objective, which is of great significance for guiding individualized treatments and evaluating prognoses.

The standard treatment for glioma includes surgery, radiotherapy, and TMZ-based chemotherapy. Surgery is the basis of the standard treatment of glioma, which is closely related to the prognoses of patients. The research by Sanai et al. [7] indicated that for patients with newly diagnosed GBMs, aggressive EOR equated with an improvement in overall survival. Hardesty et al. [8] reviewed every major peer-reviewed clinical publication from 1990 to 2012 on the role of EOR in glioma outcome and concluded that more extensive surgical resections weres associated with longer life expectancies for both low- and high-grade newly diagnosed gliomas. A meta-analysis of the association between the EOR and outcome of patients with glioblastoma (GBM), which comprised 41117 unique patients, showed that gross total resection substantially improved OS and PFS, compared with subtotal resection [10]. In our study, EOR was strongly associated with PFS and OS in HGG patients receiving standard treatment, consistent with the literature. Glioma shows invasive growth, and the tumor boundary is generally difficult to judge by conventional radiology. Therefore, the resection scope of a tumor is often limited to the tumor boundary indicated by a preoperative radiological examination, rather than the accurate histopathological boundary, which may be one of the important reasons for the easy recurrence of glioma after resection [27]. Therefore, a more extensive and more thorough resection of a tumor within the safety range can fundamentally reduce the probability of tumor recurrence, improve patient prognosis, and prolong patient survival.

A clinical trial by Stupp et al. [28] showed that patients with GBM who received concurrent TMZ and radiotherapy followed by 6 cycles of TMZ adjuvant chemotherapy had a median survival of 14.6 months and a 5-year survival rate of 9.8%. This study was a milestone in the development of glioma therapy. Many researchers also suggested that the cycle of TMZ adjuvant chemotherapy should be extended to 12 cycles [29]. A recent meta-analysis of the number of adjuvant TMZ cycles in newly diagnosed GBM, which consisted of 882 patients (461 patients for the standard chemotherapy group and 421 patients for the extended chemotherapy group), demonstrated that the extended TMZ regimen was associated with a non-significant improvement in PFS without a corresponding improvement in OS [30]. A prospective, randomized, multicenter phase II clinical trial (GEINO 14-01) compared the effect of using the standard regimen with long-cycle TMZ adjuvant chemotherapy on GBM patients and concluded that there was no statistical difference between the two treatment regimens in terms of six-month progression-free survival (PFS-6), PFS, and OS [31]. However, the study by Roldán et al. [32] showed that the median OSs of the TMZ long-cycle regimen group and the standard regimen group were 24.6 months and 16.5 months, respectively, and the difference was statistically significant (*p* < 0.05). In our study, there was no significant difference in the PFS and OS between patients receiving long-cycle and standard regimens (*p* > 0.05). Hence, the necessity of long-cycle TMZ adjuvant chemotherapy requires verification by more large-scale, multi-centered, and prospective studies.

IDH is a key rate-limiting enzyme in the tricarboxylic acid cycle, which catalyzes the oxidative decarboxylation of isocitrate to generate α-ketoglutarate and CO2, providing energy for cellular metabolism and precursors for biosynthesis [17]. IDH mutations are common in astrocytoma, oligodendroglioma, and secondary GBM [33]. The review by Śledzińska et al. [34] indicated that for adult patients, IDH mutations were positive prognostic markers and had the greatest prognostic significance. Chen et al. [35] found that the median survival time of glioma patients with IDH mutations was significantly higher than that of those without mutations, which was positively correlated with the survival rate, and the positive rate of IDH mutation decreased significantly from LGG to HGG. Some studies also demonstrated that patients with IDH-mutant primary GBM who received postoperative radiotherapy and chemotherapy had a longer PFS and OS [36,37]. In our study, patients with IDH mutation had a longer PFS and OS than those without IDH mutation, and the difference was statistically significant. We supposed that IDH-mutant HGGs may have higher tumor resection rates and may be more sensitive to postoperative radiotherapy and chemotherapy, which effectively prolonged the survival times of patients. 

MGMT is a DNA repair enzyme and is mainly distributed in the cytoplasm and repairs DNA to maintain the stability of the genome in cells [38]. In normal tissues, the CpG site in the MGMT promoter region is generally in an un-methylated state, but with the occurrence of a tumor, the promoter region is methylated. If the MGMT promoter is methylated, it will cause a loss in MGMT expression, resulting in a decrease in DNA repair and making gliomas more sensitive to chemotherapy drugs such as TMZ [39], and, therefore, the MGMT promoter methylation status was considered as an independent predictor of prognosis in patients with gliomas [40]. A meta-analysis comprising fourteen studies with 1231 GBM patients showed a significant association of MGMT methylation with a better OS with a pooled hazard ratio of 1.66 [41]. Schaff et al. [42] retrospectively identified 54 adult patients with newly diagnosed resected GBM and found that MGMT promoter methylation was statistically significantly associated with PFS and OS. The review by Binabaj et al. [43] indicated that GBM patients with MGMT methylation were associated with longer OS, although this effect was not detected for PFS. In our center, we generally recommend Stupp protocol for HGG patients regardless of whether they have MGMT promoter methylation. However, in this study, we did not find that HGG patients with MGMT promoter methylation had statistically improved PFS and OS compared with those without methylation (*p* > 0.05). We opined that this issue needed to be validated with a larger, multi-center patient sample size, which we will continue to explore in future studies. 

TERT promoter mutation is one of the common genetic mutations in adult diffuse gliomas and usually occurs in the promoter region of -124 and -146 base pairs (C228T and C250T), which can enhance TERT transcription [44]. It is essential to note that the prognostic impact of TERT promoter mutation is bivalent according to the IDH status and histological grade. In the latest WHO classification of tumors of the CNS, GBM contains only IDH-wildtype tumors, and IDH-mutant GBM was no longer defined as GBM but defined as astrocytoma, IDH mutation of the CNS (WHO 4). In general, TERT promoter mutations confer survival benefits in patients with IDH-mutant gliomas, while they are negative prognosticators in those with IDH-wildtype tumors [45]. In grade three gliomas with IDH mutations, several studies have reported that TERT promoter mutations are associated with favorable outcomes [46]. Several independent studies have reported the negative impact of TERT promoter mutations on survival in IDH-wildtype GBM cases [47,48,49]. TERT promoter status is generally stable between primary and recurrent tumor tissues in adult-type diffuse gliomas and plays an important role in the very early stages of tumor development in GBMs. In this study, no significant correlation was found between TERT promoter mutation and the survival of patients (PFS and OS). However, we found that patients with IDH and TERT promoter co-mutations had better prognoses (*p* < 0.05), which was similar to previous studies [50,51,52,53]. Hence, for the prognosis stratification of HGG patients, any single indicator may not be able to make a good judgment, and the combination of multiple indicators is more conducive to prognosis stratification [54].

The EI represented the degree of the peritumoral brain edema (PTBE) compared with tumor volume, with an index of 1.0, indicating no PTBE development, and was used only in clinical studies related to previous meningioma [55], which has not been applied to the prognosis evaluation of glioma. However, PTBE was a common feature of glioma, especially HGG [56]. Postoperative pathologically confirmed gliomas often entered PTBE beyond the tumor margin visible on radiology, so this area was often the site of tumor recurrence [57,58]. The PTBE of gliomas showed hyper-intensity on the T2WI and FLAIR sequences but no enhancement on the CE-T1WI sequence, suggesting vascular edema and tumor infiltration near the tumor. Previous studies have suggested that the degree of PTBE was related to the PFS of patients, and the more severe the PTBE was, the worse the prognosis was [59,60]. In this study, we found that EI was associated with the PFS of HGG patients receiving standard treatment, and this was the first report on the relationship between EI and PFS in HGG patients. In addition, it was indicated that age was related to the OS of HGG patients receiving standard treatment. A poor physical condition and immunity, a high degree of malignancy of the tumor, and a decline in multiple organs’ functions may be important reasons for the poor prognoses of elderly patients. 

Based on the research results, we have established free online prediction websites for PFS (https://glioma.shinyapps.io/survival_prediction_tool/, access data: 10 November 2022) and OS (https://glioma.shinyapps.io/survival_prediction_tool_os/, access data: 10 November 2022), respectively, and neurosurgeons can log in anytime and anywhere through computers or mobile phones. After entering patients’ relevant data, they can obtain prediction information associated with HGG patients’ survival. Grasping this information will assist neurosurgeons in optimizing clinical management and treatment strategies and improving the prognoses of patients to a certain extent. The predictive nomograms established in previous studies [4,61,62,63] could not automatically calculate survival time and could not realize the visualization of predicting results, but the current nomogram models can easily implement these functions.

However, our study still has some inevitable limitations. First, this was a retrospective study and not a randomized trial, lending to its inherent limitations. Secondly, the molecular detection information was not complete and did not include 1p/19q co-deletion, ATRX mutation, EGFR amplification, etc., but this molecular information was of great significance to the diagnosis and treatment of glioma, so it needs to be improved in future research. Our cohort included 35 patients with IDH1-mutated grade three gliomas which have a better prognosis and may cause a potential bias. We included this group of gliomas because in China they receive the same treatment as non-mutated IDH1 gliomas. Moreover, the strict exclusion criteria for this study resulted in a relatively small number of eligible patients being enrolled, and multi-centered, prospective, and randomized controlled clinical research on the critical factors relevant to the prognoses of adult HGGs is required to be carried out.

## 5. Conclusions

In this study, age, EOR, and IDH mutation were independent predictors for OS in HGG patients, while age, EI, EOR, and IDH mutation were independent predictors for PFS in HGG patients. In addition, patients with IDH and TERT promoter co-mutations had longer PFSs and OSs, and no apparent survival benefit was found with the long-cycle TMZ adjuvant chemotherapy compared with the standard Stupp protocol. The nomogram models were successfully developed and validated to dynamically predict the PFS and OS for HGG patients, expecting to help neurosurgeons optimize clinical management and treatment strategies. 

## Figures and Tables

**Table 1:** Baseline characteristics and results of univariate COX analyses for PFS and OS.

Variables	No. of Patients (%)	PFS				OS			
		HR	Lower 95%CI	Upper 95%CI	*p* Value	HR	Lower 95%CI	Upper 95%CI	*p* Value
Gender									
Female	119 (48.4%)	0.855	0.592	1.235	0.404	0.766	0.514	1.142	0.192
Male	127 (51.6%)								
Age									
<60	138 (56.1%)	1.186	0.804	1.750	0.390	1.330	0.729	2.427	0.353
≥60	108 (43.9%)								
KPS									
60	63 (25.6%)	1.007	0.982	1.033	0.599	0.986	0.959	1.014	0.330
70	121 (49.2%)								
80	62 (25.2%)								
Extent of resection									
Total	95 (38.6%)	1.373	1.111	1.697	0.003	1.305	1.036	1.643	0.024
Subtotal	73 (29.7%)								
Partial	78 (31.7%)								
Tumor grade									
CNS WHO 3	84 (34.1%)	1.220	0.813	1.829	0.337	0.959	0.630	1.460	0.845
CNS WHO 4	162 (65.9%)								
IDH mutation									
Negative	136 (55.3%)	0.266	0.177	0.402	<0.001	0.339	0.218	0.525	< 0.001
Positive	110 (44.7%)								
MGMT promoter methylation									
Negative	148 (60.2%)	0.515	0.345	0.769	0.001	0.609	0.400	0.928	0.021
Positive	98 (39.8%)								
TERT promoter mutation									
Negative	152 (61.8%)	0.596	0.408	0.870	0.007	0.650	0.434	0.974	0.037
Positive	94(38.2%)								
Interval between surgery and radiotherapy									
>45d	118 (48.0%)	1.169	0.810	1.689	0.404	1.305	0.875	1.945	0.192
≤45d	128 (52.0%)								
Postoperative TMZ adjuvant chemotherapy cycles									
≥9	140 (56.9%)	0.979	0.677	1.414	0.909	0.745	0.497	1.116	0.153
6	106 (43.1%)								
Tumor volume (cutoff point: 1737.15cm^3^) #									
<1737.15cm^3^	152 (61.8%)	0.913	0.622	1.342	0.645	NA	NA	NA	NA
≥1737.15cm^3^	94 (38.2%)								
Tumor volume (cutoff point: 1419.04cm^3^) #									
<1419.04cm^3^	119 (48.4%)	NA	NA	NA	NA	0.903	0.595	1.370	0.631
≥1419.04cm^3^	127 (51.6%)								
Maximum diameter (cutoff point: 1.64cm) #									
<1.64cm	105 (42.6%)	1.139	0.734	1.768	0.562	NA	NA	NA	NA
≥1.64cm	141 (57.4%)								
Maximum diameter (cutoff point: 1.37cm) #									
<1.37cm	74 (30.1%)	NA	NA	NA	NA	1.188	0.739	1.910	0.478
≥1.37cm	172 (69.9%)								
Edema index									
<3.09	125 (50.8%)	1.582	1.005	2.490	0.047	1.353	0.896	2.043	0.151
≥3.09	121 (49.2%)								
T1WI									
Hypo-intensity	199 (80.9%)	1.043	0.756	1.440	0.796	0.806	0.538	1.208	0.296
Iso-intensity	39 (15.9%)								
Hyper-intensity	8(3.2%)								
T2WI									
Hypo-intensity	6 (2.5%)	0.925	0.584	1.465	0.740	0.925	0.565	1.515	0.756
Iso-intensity	33 (13.4%)								
Hyper-intensity	207 (84.1%)								
Flair									
Hypo-intensity	5 (2.0%)	0.754	0.596	0.488	0.202	0.700	0.443	1.105	0.126
Iso-intensity	30 (12.2%)								
Hyper-intensity	211 (85.8%)								
DWI									
Hypo-intensity	4 (1.6%)	0.717	0.465	1.106	0.132	0.659	0.417	1.039	0.728
Iso-intensity	7 (2.8%)								
Hyper-intensity	235 (95.6%)								
Enhanced pattern									
Heterogenous	222 (90.2%)	0.878	0.492	1.569	0.661	1.178	0.677	2.050	0.562
Homogenous	24 (9.8%)								

Footnote: # different cut-off points were calculated for the analyses of PFS and OS. Abbreviations: PFS—progression-free survival; OS—overall survival; HR—hazard ratio; 95%CI—95% confidence interval; KPS—Karnofsky performance status; TMZ—temozolomide; NA—not applicable.

## Data Availability

The data presented in this study are available on request from the corresponding author. The data are not publicly available due to protecting patients’ privacy.

## Supplementary Materials

The following supporting information can be downloaded at: https://www.mdpi.com/article/10.3390/jcm12010196/s1. Figure S1: The optimal cut-off points for edema index (A), maximum diameter (B), and tumor volume (C) by running log-rank test for PFS. Figure S2. The optimal cut-off points for edema index (A), maximum diameter (B), and tumor volume (C) by running log-rank test for OS. Figure S3. The calibration curves of PFS for training samples (A-C) and validation samples (D-F) at 12, 24 and 48 months. Figure S4. Time-dependent AUC curves of PFS for training (A) and validation (B) samples. Figure S5. The calibration curves of OS for training samples (A-C) and validation samples (D-F) at 12, 24 and 48 months. Figure S6. Time-dependent AUC curves of OS for training (A) and validation (B) samples.
