# Supplementary material for: Nomogram Model for Predicting the Prognosis of High-Grade Glioma in Adults Receiving Standard Treatment: A Retrospective Cohort Study
Source: J Clin Med. 2022 Dec 27;12(1):196. doi: 10.3390/jcm12010196 (PMC9821755; doi:10.3390/jcm12010196)
Supplement: Supplementary file 1 [file jcm-12-00196-s001.zip › jcm-2063431-supplementary.pdf]

Journal of
Clinical Medicine

Supplementary Material

Supplementary Figure Legends...1

Figure S1. The optimal cut-off points for edema index (A), maximum diameter (B), and tumor volume (C) by running log-rank test for PFS...2

Figure S2. The optimal cut-off points for edema index (A), maximum diameter (B), and tumor volume (C) by running log-rank test for OS 3

Figure S3. The calibration curves of PFS for training samples (A-C) and validation samples (D-F) at 12, 24 and 48 months 4

Figure S4. Time-dependent AUC curves of PFS for training (A) and validation (B) samples...5

Figure S5. The calibration curves of OS for training samples (A-C) and validation samples (D-F) at 12, 24 and 48 months...6

Figure S6. Time-dependent AUC curves of OS for training (A) and validation (B) samples...7

Supplementary Figure Legends

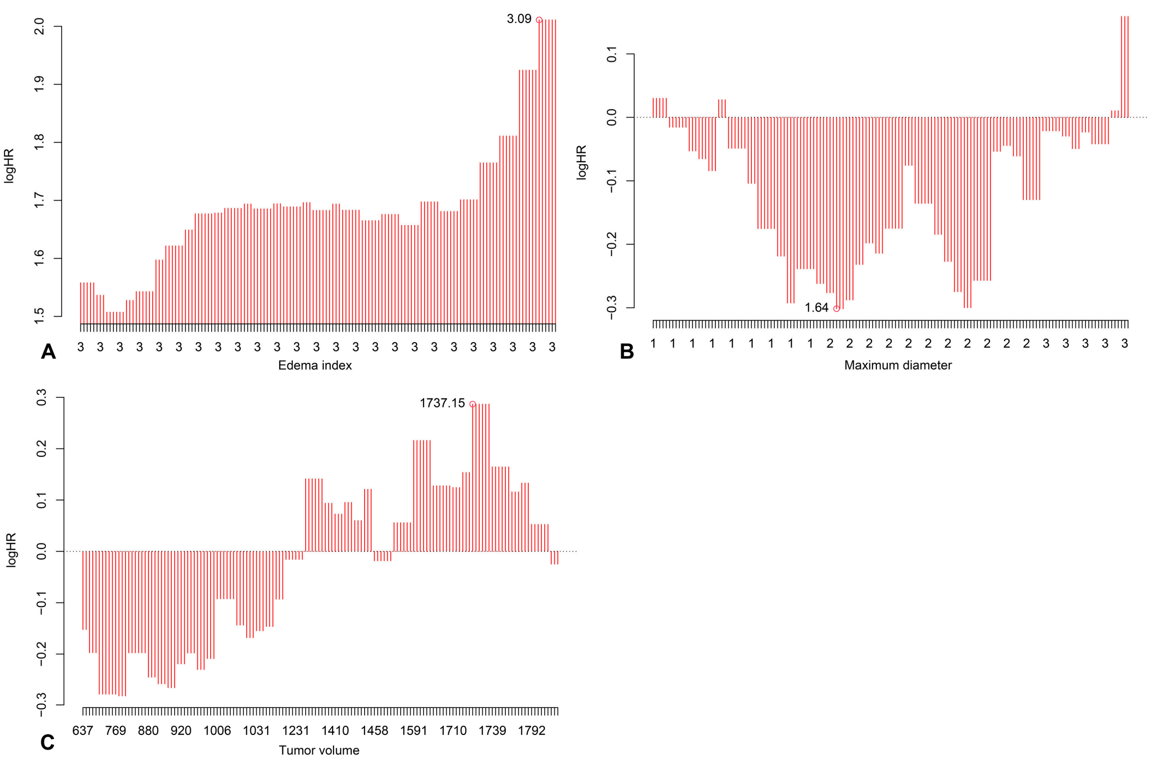

Figure S1. The optimal cut-off points for edema index (A), maximum diameter (B), and tumor volume (C) by running log-rank test for PFS. The optimal point was defined as the point with the most higher absolute logHR. As a result, the optimal cut-off points were 3.09, 1.64 cm, and 1737.15 cm^3 for the three continuous predictors.

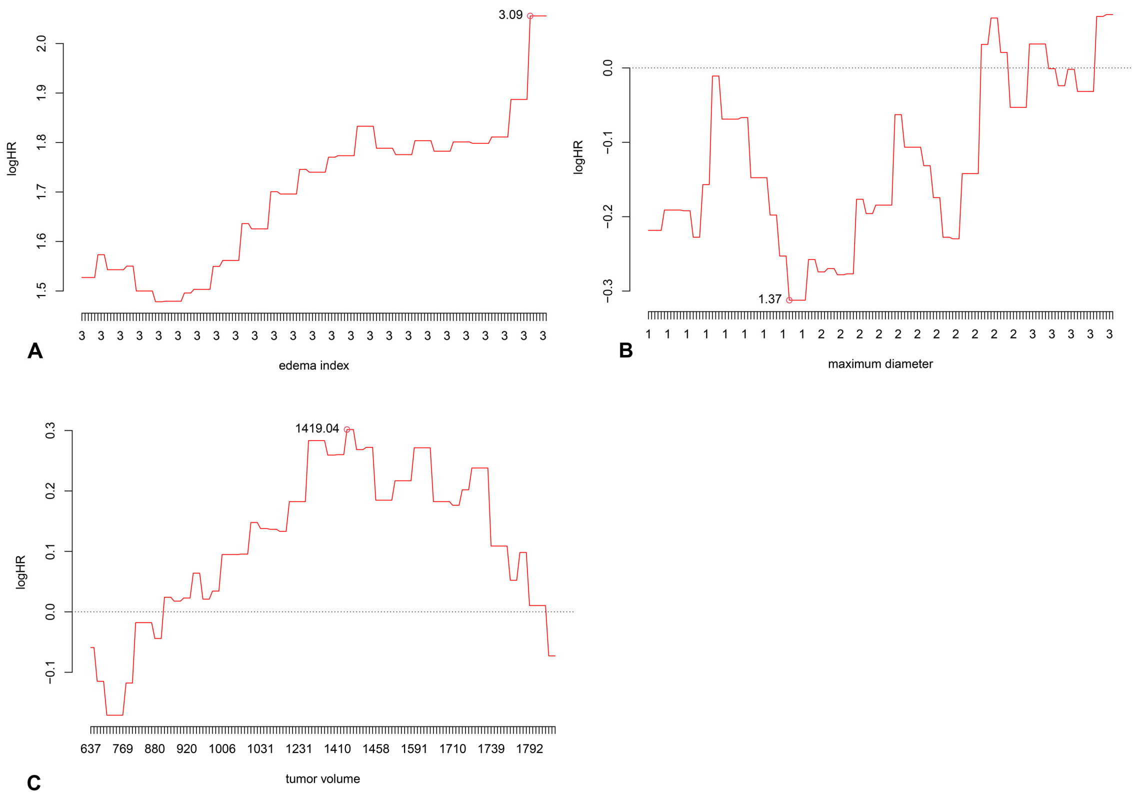

Figure S2. The optimal cut-off points for edema index (A), maximum diameter (B), and tumor volume (C) by running log-rank test for OS. The optimal point was defined as the point with the most higher absolute logHR. As a result, the optimal cut-off points were 3.09, 1.37 cm, and 1419.04 cm^3 for the three continuous predictors.

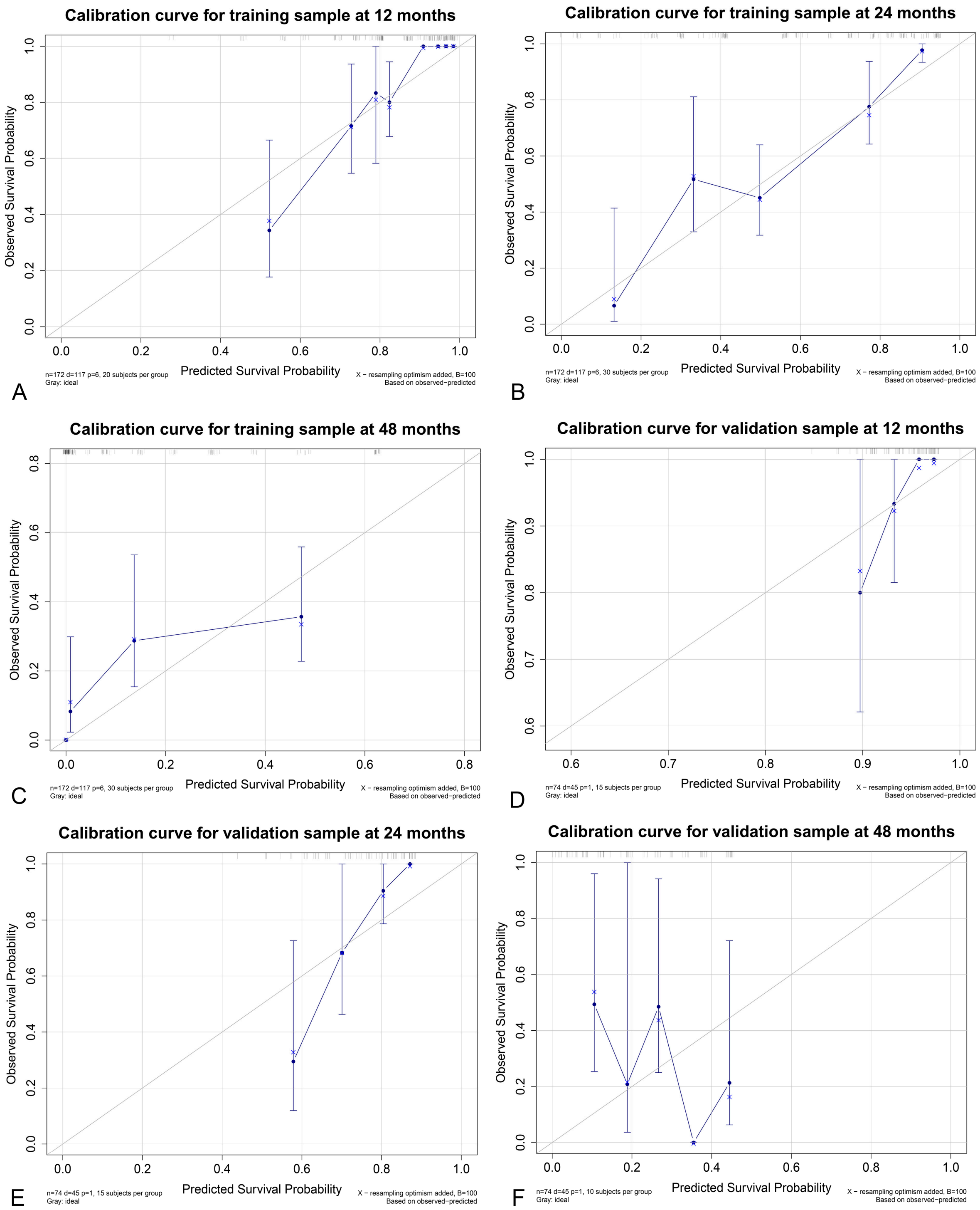

Figure S3. The calibration curves of PFS for training samples (A-C) and validation samples (D-F) at 12, 24 and 48 months. The curves presented favorable consistency between the predicted and actual PFS.

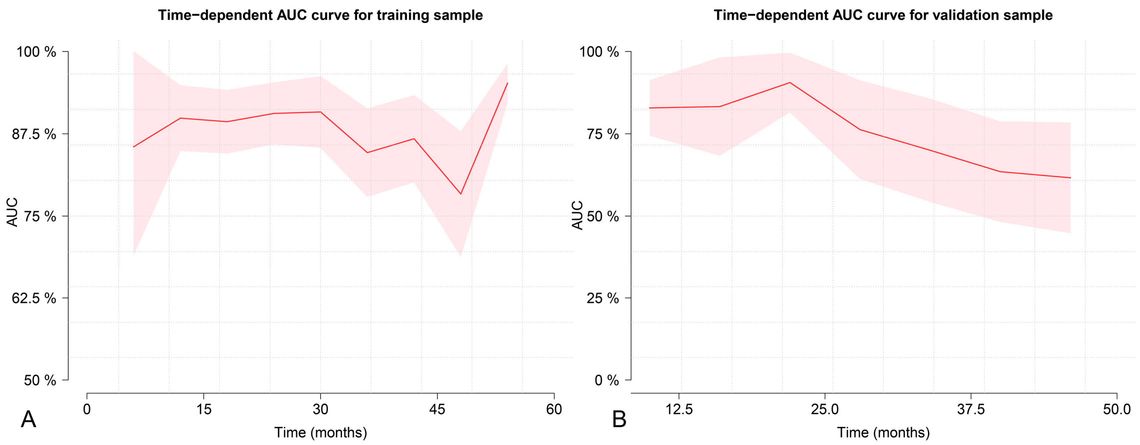

Figure S4. Time-dependent AUC (area under ROC curve) curves of PFS for training (A) and validation (B) samples. This model showed favorable discrimination ability in both training and validation samples.

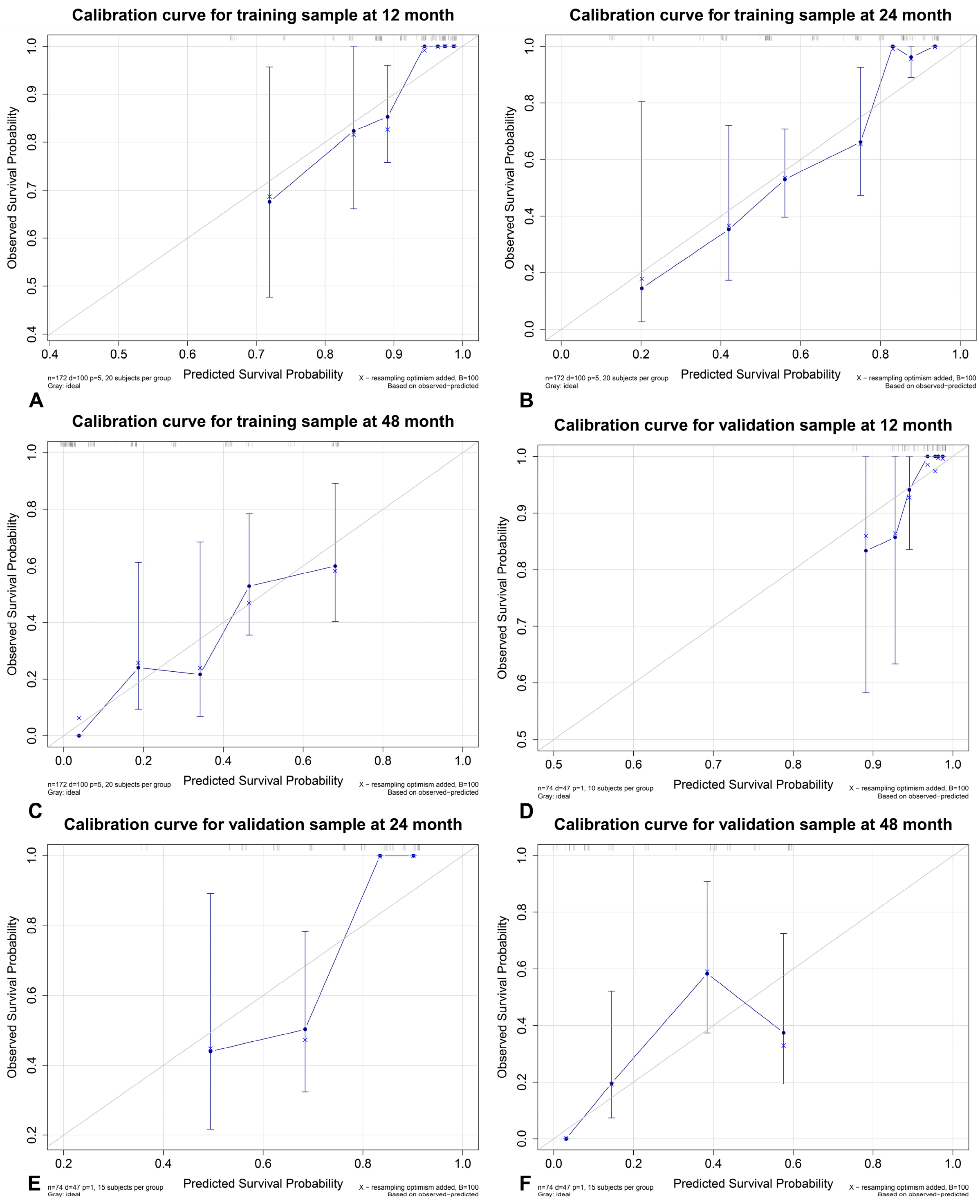

Figure S5. The calibration curves of OS for training samples (A-C) and validation samples (D-F) at 12, 24 and 48 months. The curves presented favorable consistency between the predicted and actual OS.

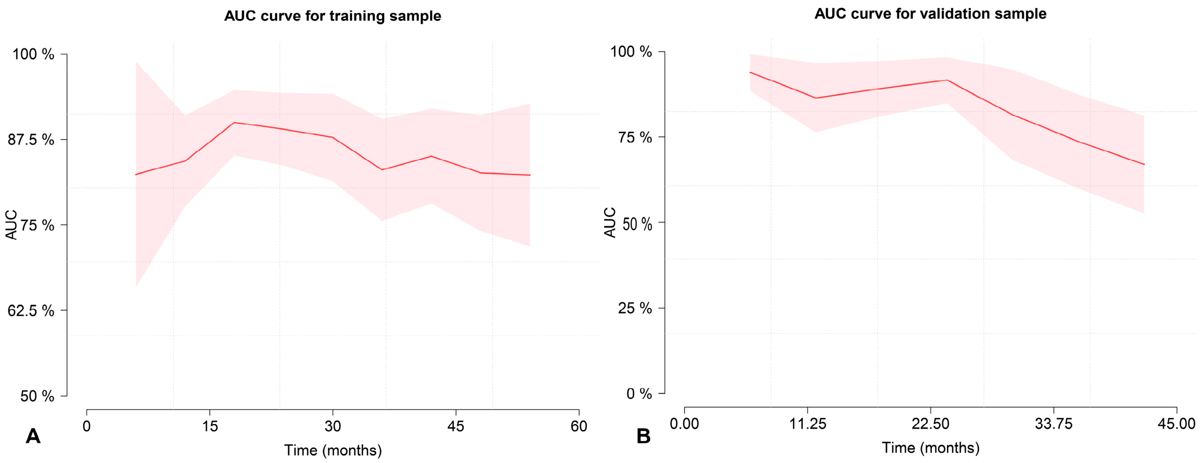

Figure S6. Time-dependent AUC (area under ROC curve) curves of OS for training (A) and validation (B) samples. This model showed favorable discrimination ability in both training and validation samples.
